# Green Approach to Enhance Dissolution of Gliclazide: Thermoresponsive Solid Dispersion Based on Poloxamer 188/Propylene Glycol/Labrasol Ternary System

**DOI:** 10.3390/pharmaceutics18060702

**Published:** 2026-06-08

**Authors:** Abdelrahman Y. Sherif, Mohamed A. Ibrahim

**Affiliations:** Department of Pharmaceutics, College of Pharmacy, King Saud University, Riyadh 11451, Saudi Arabia; mhamoudah@ksu.edu.sa

**Keywords:** thermoresponsive solid dispersion, poloxamer 188, gliclazide, labrasol, I-optimal mixture design

## Abstract

**Background/Objectives:** Gliclazide’s limited water solubility restricts its absorption across the gastrointestinal tract and compromises its therapeutic performance. This study developed a thermoresponsive solid dispersion based on the inverted thermoresponsive behavior of poloxamer 188 in propylene glycol. **Methods:** A solubility study was conducted to select components for the thermoresponsive solid dispersion. An I-optimal mixture design was used to optimize the concentrations of the thermoresponsive solid dispersion components (poloxamer 188, propylene glycol, and labrasol). FTIR and XRD were used to investigate the mechanism underlying the inverted thermoresponsive behavior. Finally, the influence of the thermoresponsive solid dispersion on gliclazide dissolution was evaluated through in vitro dissolution testing. **Results:** Surfactant screening identified labrasol as the optimal surfactant owing to a superior increase in gliclazide solubility compared to propylene glycol alone (2.29-fold). The optimized thermoresponsive solid dispersion (poloxamer 188, propylene glycol, and labrasol at 13.89, 21.43, and 64.68% *w*/*w*, respectively) achieved a drug solubility of 10.68 mg/g and a phase transition temperature of 36 °C. XRD and FTIR confirmed that hydrogen bonding is responsible for the system’s conversion between the solid and liquid states. Compared with raw gliclazide, the optimized formulation demonstrated an 8.4-fold increase in the initial dissolution rate and significantly improved dissolution efficiency from 21.77 ± 4.74% to 74.85 ± 2.33%. **Conclusions:** The present thermoresponsive solid dispersion provides a green alternative to conventional solid dispersion techniques. It avoids reliance on organic solvents, processing that demands high energy input, and additional post-processing operations.

## 1. Introduction

As a second-generation sulfonylurea, gliclazide mediates its hypoglycemic activity through stimulation of pancreatic β-cells [1]. Beyond its glucose-lowering effect, gliclazide possesses a distinctive azabicyclooctyl ring that exhibits potent free radical scavenging activity. This affords protection to β-cells against oxidative damage [2]. Moreover, gliclazide demonstrates beneficial effects on the vascular system, including reductions in platelet reactivity and amelioration of endothelial oxidative stress [3,4]. These multifaceted therapeutic benefits have contributed to gliclazide’s inclusion on the World Health Organization’s List of Essential Medicines [5]. Despite these clinical advantages, the pharmaceutical utility of gliclazide is hampered by its physicochemical properties. The reported low aqueous solubility of gliclazide (0.19 mg/mL) negatively affects its dissolution in the gastrointestinal tract. This results in a poor therapeutic response following oral administration [6]. Therefore, this solubility-limited bioavailability necessitates the development of formulation strategies that enhance gliclazide’s dissolution performance to achieve predictable antihyperglycemic activity.

Among the various approaches investigated to address limited solubility, the solid dispersion approach has been widely studied. The dispersion of hydrophobic drugs within a polymeric carrier enhances their bioavailability through various mechanisms. The hydrophilic carrier polymers embedded within the matrix enhance the wettability of the dispersed drug [7,8,9]. Moreover, converting a drug from its crystalline form into an amorphous one promotes its dissolution in the aqueous environment of the intestine [10,11]. Finally, the reported surfactant-like activity of the used polymer keeps the drug in a solubilized state and prevents its precipitation after its solubilization [12,13,14].

Previously reported studies have shown that gliclazide solid dispersions prepared using various techniques enhance solubility and dissolution. However, several limitations exist with these conventional approaches. For example, Lu et al. implemented a spray-drying approach to formulate gliclazide amorphous solid dispersions, using hydroxypropyl methylcellulose acetate succinate dissolved in acetone as the carrier [15]. In addition, Febriyenti et al. utilized the solvent evaporation method with polyvinylpyrrolidone K-30 and polyethylene glycol 6000 as carriers dissolved in ethanol [16]. However, the demand for organic solvents in both spray drying and solvent evaporation techniques diminishes their eco-friendliness and raises concerns about residual solvents in the final product. To overcome this limitation, Mansour and Aly prepared a solid dispersion from an aqueous solution via lyophilization without using an organic solvent. Unfortunately, the inherent prolonged lyophilization processing limits its suitability for industrial-scale production [17]. Alternatively, Huang et al. used hot-melt extrusion as a solvent-free continuous manufacturing approach to prepare gliclazide amorphous solid dispersions. However, the inherent thermal instability of gliclazide, as indicated by the measured degradation percentage (5%), underscores the persistent challenge of processing via hot-melt extrusion [18]. In addition to the aforementioned limitations of the approaches used, the resultant solidified mass requires post-processing steps, including pulverization and sieving, to obtain a uniform particle-size powder. Therefore, there remains a pressing need for a green solid dispersion approach that eliminates the use of organic solvents, avoids energy-intensive instruments, and requires no post-processing steps.

In response to these challenges, the present study introduces a thermoresponsive solid dispersion platform that exploits the inverted thermoresponsive behavior of poloxamer in non-aqueous solvents. Normally, an aqueous system containing a poloxamer polymer undergoes a transition from liquid to solid state when exposed to high temperature [19]. On the contrary, it has been reported that replacing water with a non-aqueous solvent reverses the thermal transition behavior. For example, Fahad et al. demonstrated that media containing non-aqueous solvent modify the thermoresponsive behavior of poloxamers [20]. This thermoresponsive paradigm offers a compelling advantage for oral solid dispersion formulations. It resolves the aforementioned limitations of previously reported approaches and provides a new avenue for industrial application of solid dispersion. In addition, the use of a pharmaceutically accepted, generally recognized as safe-listed non-aqueous excipient solvent, such as propylene glycol, addresses the limitation of using toxic organic solvents, such as ethanol [21,22].

Thus, the present study aimed to develop a thermoresponsive solid dispersion of gliclazide. The platform exploits the inverted thermoresponsive behavior of poloxamer 188 in propylene glycol. This yields a system that is solid at room temperature and liquefies at body temperature. The novelty of this approach lies in three features that overcome the main drawbacks of conventional solid dispersion techniques. These include avoiding the use of organic solvents, using only mild thermal processing, and requiring no post-processing steps. An I-optimal mixture design was applied to optimize the ternary composition of poloxamer 188, propylene glycol, and labrasol with respect to drug solubility and phase transition temperature. The mechanism governing the solid-to-liquid thermal transition of the formulation was elucidated using Fourier transform infrared (FTIR) spectroscopy and X-ray diffraction (XRD). Finally, the dissolution performance of the optimized thermoresponsive solid dispersion was compared with that of raw gliclazide to demonstrate the pharmaceutical superiority of this approach.

## 2. Materials and Methods

### 2.1. Materials

Gliclazide was kindly provided by Saudi Pharmaceutical Industries and Medical Appliances Corp. (Qassim, Saudi Arabia). Propylene glycol was supplied from Winlab Laboratory (Market Harborough, UK). HCO-30 (polyoxyl 30 hydrogenated castor oil) and TO-10V were obtained from Nikko Chemicals Co., Ltd. (Tokyo, Japan). Kolliphor EL was provided by BASF (Ludwigshafen, Germany). Labrasol (caprylocaproyl macrogol-8 glycerides) was kindly provided by Gattefossé (Saint-Priest, France). Tween 80 and Tween 20 were supplied by ALPHA CHEMIKA (Mumbai, India) and BDH (Poole, UK), respectively.

### 2.2. Ultra-Performance Liquid Chromatography (UPLC) for Gliclazide Analysis

Gliclazide content in both saturation-solubility samples and dissolution aliquots of the thermoresponsive solid dispersion was assayed by reversed-phase ultra-performance liquid chromatography (UPLC). Separations were performed using a Dionex UltiMate 3000 instrument (Thermo Fisher Scientific, Sunnyvale, CA, USA) consisting of quaternary pumping, an automatic sample injector, a temperature-controlled column oven, and photodiode-array detection. A BEH C18 column (50 × 2.1 mm, 1.7 µm particle size; Waters Corporation, Milford, MA, USA) was held at 25 ± 0.1 °C, and elution was performed isocratically with a 60:40 (*v*/*v*) blend of 0.1% aqueous formic acid solution and acetonitrile pumped at 0.4 mL/min. The injection volume was 2 µL, and the total run time was 2.4 min. Detection wavelength was set to the drug’s UV maximum at 228 nm. For calibration, a solution containing 500 µg/mL gliclazide was diluted to prepare seven standards spanning 1–75 µg/mL (1, 2, 5, 10, 20, 50, and 75 µg/mL). Linearity was assessed by regressing the integrated peak area against the nominal concentration.

### 2.3. Solubility of Gliclazide

Gliclazide solubility was studied using a saturation solubility method to assess drug solubility in propylene glycol alone, a propylene glycol/surfactant mixture, or the thermoresponsive solid dispersion. An excess amount of gliclazide and the tested agent were placed in a 5 mL glass beaker. The mixture was stirred with an IKA magnetic stirrer (Staufen, Germany) at 1000 rpm for one day at controlled room temperature (23 ± 2 °C). The next day, the mixture was transferred to a 2 mL Eppendorf tube and centrifuged to precipitate the undissolved drug. The Eppendorf centrifuge was used for 15 min at 13,000 rpm. Drug concentration in supernatant was estimated using the developed UPLC method.

### 2.4. Experimental Design

Stat-Ease 360 software (version 13; Stat-Ease, Inc., Minneapolis, MN, USA) was utilized to optimize thermoresponsive solid dispersion components. The current study ranges for the three components were established based on the following objectives. Regarding poloxamer 188, concentrations below 5% *w*/*w* were insufficient to ensure solidification of the formulation at room temperature. On the other hand, concentrations above 15% *w*/*w* produced systems that converted to liquid only above 37 °C. Therefore, the 5–15% *w*/*w* range was selected, in which both the solid state at room temperature and the liquefaction upon exposure to body temperature are achievable simultaneously. In this regard, propylene glycol and labrasol were varied across the maximum allowable range, complementary to poloxamer 188. Therefore, 10–40% *w*/*w* was assigned to propylene glycol owing to its interaction with poloxamer, and 45–85% *w*/*w* to labrasol to achieve maximum drug loading. This was intentionally chosen to enable experimental design that fully captures the impact of the propylene glycol-to-labrasol ratio on both gliclazide solubility and the phase transition temperature. The impact of the three selected components (poloxamer 188, propylene glycol, and labrasol) on the studied responses was explored. Table 1 shows the suggested design, comprising 17 experimental runs. Solubility (mg/g) and phase transition temperature (°C) were selected as the response variables. The data was analyzed using Stat-Ease 360 (version 13). Model selection, term significance (F-tests), lack-of-fit testing, and component effect analysis (in the Cox and Piepel directions) were performed using the software. Design validation was based on three independent batches of the optimized formulation, compared with the 95% prediction intervals of the fitted models.

### 2.5. Preparation of Thermoresponsive Solid Dispersion Formulations

The thermoresponsive solid dispersion formulations recommended by Stat-Ease 360 software (Table 1) were fabricated using a straightforward mixing method. Following the proportions listed in Table 1, precise quantities of poloxamer 188, propylene glycol, and labrasol were weighed out and transferred into a 10 mL glass beaker. The mixture was stirred with a magnetic stirrer and incubated at 40 °C for 2 h to ensure complete dissolution of poloxamer 188 in the propylene glycol/labrasol mixture.

### 2.6. Phase Transition Temperature Measurement

Phase transition temperature was considered as the lowest temperature at which a given formulation switched from a rigid to a freely flowing state. This endpoint reflects the temperature window relevant to in vivo melting. Solidified samples were transferred into glass test tubes and pre-equilibrated for 5 min at 28 ± 0.1 °C in a thermostatic water bath. Following each holding period, the tubes were inspected visually for loss of structural integrity. The bath set point was raised in 1.0 ± 0.1 °C increments, and the inspection cycle was repeated until all formulations had been characterized.

### 2.7. Assessment of Inverted Thermoresponsive Behavior

The optimized thermoresponsive solid dispersion was placed in a hard gelatin capsule and left to solidify. A preheated phosphate buffer (pH 6.8) at 37 °C was placed in a cylindrical glass beaker, and the capsule was surrounded with a wire to facilitate immersion. A photograph was captured to show the liquefaction of the thermoresponsive solid dispersion upon exposure to physiological body temperature.

### 2.8. FTIR

Fourier transform infrared (FTIR) spectroscopy was employed to investigate the molecular interactions among poloxamer 188, propylene glycol, and labrasol within the thermoresponsive solid dispersion formulation. Spectra were obtained using a PerkinElmer Spectrum-100 instrument (Waltham, MA, USA) fitted with an attenuated total reflectance sampling module, and test agents were scanned from 650 to 4000 cm^−1^. Drug-free thermoresponsive solid dispersion formulations were investigated in the solid state (room temperature) and in the liquid state (heated to 40 °C).

### 2.9. XRD

X-ray analysis was performed to further elucidate the solid-state structural basis of the thermoresponsive behavior of the thermoresponsive solid dispersion. Diffractograms were collected for poloxamer 188, crystalline gliclazide, and the drug-free and drug-loaded optimized thermoresponsive solid dispersion formulations, using an Ultima IV instrument (Rigaku Inc., Tokyo, Japan) fitted with a Cu Kα source operated at 40 kV and 30 mA, a fixed U4 monochromator, and scintillation-counter detection. Samples were scanned in continuous mode over a 2θ range of 3–60° at a scan speed of 1.0°/min.

### 2.10. In Vitro Dissolution Study

The impact of thermoresponsive solid dispersion matrix on gliclazide dissolution was assessed through a comparative in vitro dissolution experiment. Tests were carried out on a USP Apparatus II paddle system (LOGAN Inst. Corp., Somerset, NJ, USA). Hard gelatin capsules containing either raw gliclazide or the optimized thermoresponsive solid dispersion containing 10 mg of gliclazide were prepared. The composition of optimized thermoresponsive solid dispersion consisted of poloxamer 188, propylene glycol, and labrasol at 13.89, 21.43, and 64.68% *w*/*w*, respectively. The ratio of excipients to drug is 99:1 (*w*/*w*) to achieve 10 mg/g drug loading. After that, the capsules were surrounded by a wire sinker to prevent them from floating during the experiment. The vessels containing 900 mL of phosphate buffer (pH 6.8) were pre-equilibrated to 37 ± 0.5 °C prior to the experiment. The paddle speed was set at 50 rpm. Aliquots were collected at 5, 10, 15, 30, 45, and 60 min and filtered in-line through a 10 µm syringe filter. Drug concentrations in the collected samples were estimated using a validated UPLC method.

The initial dissolution rate (IDR) and dissolution efficiency (DE) were calculated to provide a comprehensive assessment of the dissolution performance. The IDR was determined from Equation (1) over the initial 15 min. This offers a clear measure of how effectively the thermoresponsive solid dispersion formulation accelerates drug dissolution during the early phase. Dissolution efficiency (DE) was calculated as the ratio of the area under the dissolution curve up to 60 min to the area of the rectangle described by 100% dissolution at the same time point, as expressed in Equation (2).

IDR = Qt/t(1)

where IDR is the initial dissolution rate (%/min), Qt is the percentage of drug released at a specific time (%), and t is the time period (min).

DE (%) = (∫_0_^t^ y · dt/(y_100_ × t)) × 100 (2)

where y is the percentage of drug dissolved at time t, y_100_ is 100% dissolution, and t is the total time of the dissolution study.

### 2.11. Use of Generative AI

During the preparation of this manuscript, the authors used Claude (Anthropic, San Francisco, CA, USA; Opus 4.7) to write portions of it, including language editing and the identification of relevant literature. The authors independently verified all cited references and the accuracy of all statements against the primary sources, reviewed and edited the output, and took full responsibility for the content of this publication.

## 3. Results and Discussion

### 3.1. UPLC Method Development

The developed isocratic UPLC method was used to quantify gliclazide. As shown in Figure S1a, gliclazide eluted as a sharp and symmetrical peak at a retention time of approximately 2.14 min with a total run time of 2.4 min. The photodiode array spectrum of the gliclazide peak (Figure S1b) exhibited an absorption maximum at 228 nm. Therefore, this wavelength was selected for the quantification of gliclazide. The regression parameters were assessed at a concentration range of 1–75 µg/mL and are summarized in Table 2. The method used showed outstanding linearity with a coefficient of determination (R^2^) of 0.9994 ± 0.0005.

### 3.2. Selection of Formulation Components

Propylene glycol was selected as a non-aqueous solvent for preparing a solid dispersion containing poloxamer and the loaded drug. It is widely used in oral pharmaceutical products owing to its safety and is listed as a pharmaceutically accepted generally recognized as safe excipient [21,22]. A previous study showed that poloxamer dissolved in nonaqueous solvents undergoes thermal gelation upon cooling, which melts upon heating [20,23]. This system offers an outstanding advantage when preparing pharmaceutical formulations intended for oral administration. The transition of formulation to the semisolid state during storage prevents formulation leakage issues associated with liquid formulations. The expected liquefaction upon exposure to higher temperatures (body temperature, 37 °C) than during storage (room temperature, 25 °C) ensures complete release of the drug and excipients in the gastrointestinal tract. Therefore, the present study aims to develop a thermoresponsive solid dispersion formulation using poloxamer and propylene glycol as polymer and non-aqueous solvent, respectively.

### 3.3. Solubility of Gliclazide in Propylene Glycol

The solubility of gliclazide in propylene glycol was studied to provide a clear indication of its baseline drug-loading capacity. The data obtained showed that the solubility of gliclazide in propylene glycol was 4.59 ± 0.58 mg/g. The solubilizing power of propylene glycol may be explained by the anticipated formation of hydrogen bonds linking its hydroxyl groups to the sulfonyl and carbonyl moieties of gliclazide. These findings are consistent with earlier reports demonstrating that gliclazide engages in hydrogen bonding with solvents possessing strong hydrogen-bond acceptor capacity [24].

### 3.4. Solubility of Gliclazide in Poloxamer 188/Propylene Glycol

Poloxamer 188 was selected as the polymer for preparing the thermoresponsive solid dispersion. However, the impact of poloxamer on gliclazide solubility needs to be studied first to provide a clear indication of its impact on drug solubility. The solubilization of poloxamer 188 in a non-aqueous solvent (propylene glycol) significantly increased the gliclazide solubility to 5.78 ± 0.03 mg/g. This could be attributed to the formation of a hydrogen bond between the ether oxygen atoms within the poloxamer structure and the sulfonyl and amino groups of gliclazide [25].

### 3.5. Solubility of Gliclazide in Surfactant

The addition of a surfactant to the proposed formulation was intended to increase the drug-loading capacity of the thermoresponsive solid dispersion. A screening solubility study was conducted to select an appropriate one with the aforementioned benefit. The current results showed that the addition of surfactant to propylene glycol increases the solubility of gliclazide by 1.53- to 2.29-fold, as presented in Table 3. The solubilization capacity of gliclazide within propylene glycol/surfactants mixtures followed the rank order: labrasol > Tween 80 > TO-10V > Tween 20 > Kolliphor EL > HCO-30. Therefore, labrasol was selected as the optimal surfactant to prepare the thermoresponsive solid dispersion formulation.

In this regard, the three components, namely propylene glycol, poloxamer 188, and labrasol, were selected as optimum components for the preparation of the thermoresponsive solid dispersion. However, Stat-Ease 360 software (version 13) was used to give a comprehensive overview of the impact of components on formulation behavior and to select the optimum component ratio.

### 3.6. Experimental Design and Model Selection

**Figure 1:**
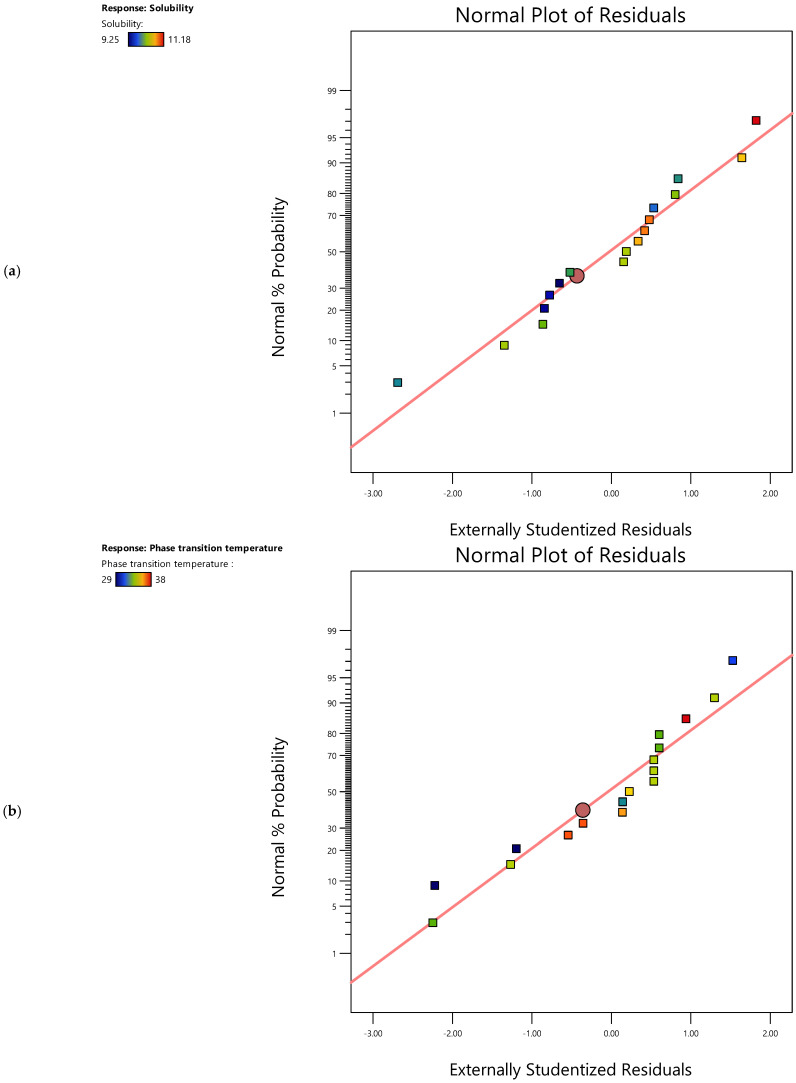
Normal probability plots of externally studentized residuals for (**a**) solubility and (**b**) phase transition temperature responses.

Stat-Ease 360 software was used to explore the impact of the ratios of the thermoresponsive solid dispersion components on the measured responses. The I-optimal mixture design yielded 17 formulations, and the measured responses, including solubility and phase transition temperature, are presented in Table 4. The appropriate polynomial model for each response was selected using sequential model selection based on sums of squares, lack-of-fit tests, and model summary statistics. The statistical criteria for model selection are summarized in Table 5. For the solubility response, the quadratic model was identified as the best-fitting model based on a significant *p*-value (0.0201), a non-significant lack-of-fit *p*-value (0.4009), an adjusted R^2^ of 0.5097, and a predicted R^2^ of 0.2241. For the phase transition temperature response, the linear model demonstrated a good fit with a highly significant *p*-value (<0.0001), a non-significant lack of fit (*p* = 0.2846), an adjusted R^2^ of 0.8868, and a predicted R^2^ of 0.8440. The non-significant lack-of-fit values for both models confirm that the models adequately fit the experimental data without systematic deviation. In addition, Figure 1 shows the alignment of data points along the diagonal reference line, confirming the normal distribution of residuals and validating the adequacy of the selected models.

### 3.7. Solubility

**Figure 2:**
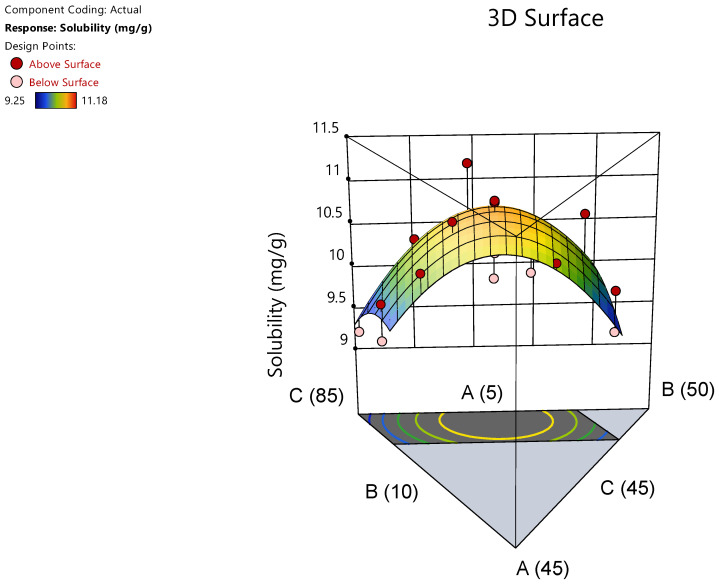
Three-dimensional response surface plot showing the effect of (A) poloxamer, (B) propylene glycol, and (C) labrasol components within the thermoresponsive solid dispersion formulation on gliclazide solubility (mg/g).

The solubility of gliclazide in the prepared thermoresponsive solid dispersion formulations ranged from 9.25 to 11.18 mg/g (Table 4). The three-dimensional response surface plot presented in Figure 2 shows the quadratic relationship between formulation composition and gliclazide solubility. Moreover, the Analysis of variance (ANOVA) results of the solubility study based on the fitted quadratic model are presented in Table 6. The results show that the interaction between propylene glycol and labrasol (BC) was the only statistically significant term (*p* = 0.0013). This finding indicates the synergistic interaction between propylene glycol and labrasol exerts a dominant influence on gliclazide solubility.

The fitted quadratic model predicted a dome-shaped response surface (Figure 2) with maximum gliclazide solubility located in the interior of the experimental region. The highest solubility was observed at a labrasol-to-propylene glycol ratio of approximately 2.75:1. This finding indicates that this ratio is favorable to enhance drug solubility within the thermoresponsive solid dispersion formulation. Gliclazide solubility was significantly decreased when the ratio of either component was increased relative to the other one. This finding suggests a complementary solubilization mechanism by both excipients. Propylene glycol could enhance the solubilization of gliclazide through hydrogen-bond interactions between the hydroxyl groups of propylene glycol and the sulfonyl and carbonyl groups of gliclazide [24,26]. Similarly, the acylglycerol portion of labrasol provides a lipophilic moiety that could be responsible for the solubilization of the aromatic tolyl moiety of gliclazide [22,27]. Although labrasol dominates by weight, it has a higher molecular weight than propylene glycol. As a result, propylene glycol molecules outnumber labrasol molecules, which showed the highest drug solubility (Run 10). The demand for more propylene glycol molecules supports its role as the primary hydrogen-bond donor in drug solubilization. Therefore, any deviation from this optimal ratio of the two agents negatively affects the solubilization of gliclazide in the formulation.

### 3.8. Phase Transition Temperature

**Figure 3:**
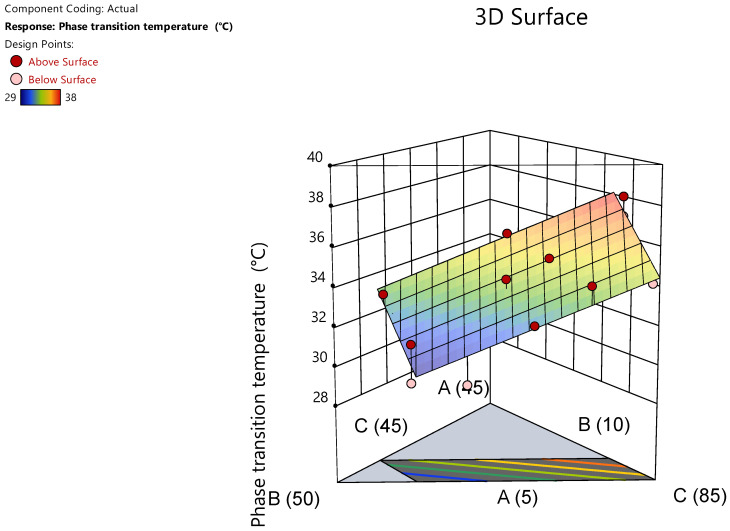
Three-dimensional response surface plot showing the effect of (A) poloxamer, (B) propylene glycol, and (C) labrasol components within the thermoresponsive solid dispersion formulation on phase transition temperature (°C).

The phase transition temperature of the thermoresponsive solid dispersion formulations ranged from 29 to 38 °C (Table 4). The ANOVA results for phase transition temperature indicated that the linear model fit was significant (*p*-value < 0.0001). The component effects analysis (Table 7) revealed that poloxamer 188 and propylene glycol significantly contributed to the measured phase transition temperature. The data obtained showed that propylene glycol exerted a stronger impact than poloxamer 188, as indicated by the Cox values (−6.42 and +4.15, respectively). However, labrasol had no significant effect on the measured phase transition temperature (*p* = 0.6020). Moreover, the three-dimensional response surface plot presented in Figure 3 shows the linear relationship between formulation composition and phase transition temperature.

Measurement of phase transition temperature is necessary to ensure that formulation compositions can convert to a liquid state at physiological body temperature. According to the literature, poloxamer 188 can form hydrogen bonds with propylene glycol, leading to solid matrix formation [23]. The observed opposing effects of poloxamer 188 and propylene glycol may be related to their functional roles in the thermoresponsive solid dispersion system. The observed positive impact of poloxamer 188 concentration may be due to its longer chain length relative to propylene glycol. Therefore, increasing the concentration of the former increases the network structure of the thermoresponsive solid dispersion matrix formed. This agrees with a previous study that showed a correlation between the size of matrix-forming agents and the strength of the formed matrix [28]. Conversely, the negative impact of propylene glycol could be attributed to its small size, which reduces the intermolecular network strength compared to the long poloxamer polymer. Finally, the absence of a labrasol effect on the phase transition temperature could result from its lack of interaction with the matrix formed within the thermoresponsive solid dispersion. Furthermore, the current findings were extensively investigated in the following sections using FTIR and XRD.

### 3.9. Selection of Optimized Formulation

**Figure 4:**
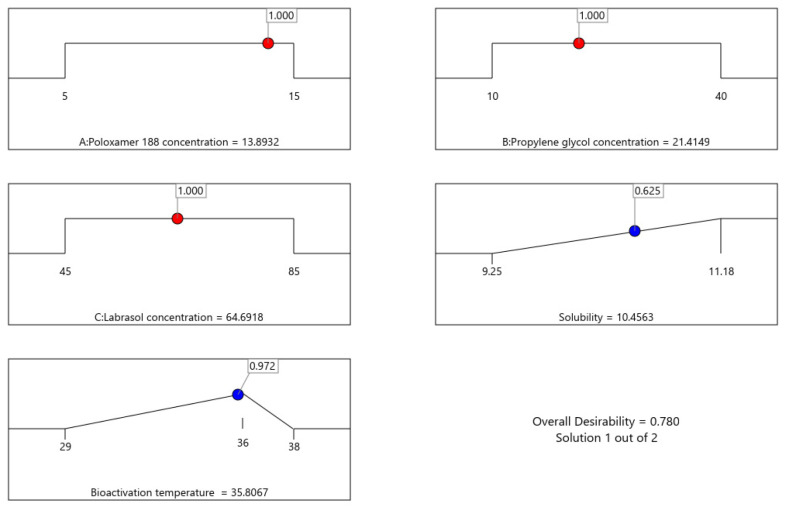
Ramp plots display the composition of the optimized thermoresponsive solid dispersion formulation, the expected response values, and overall desirability.

The composition of an optimized thermoresponsive solid dispersion was suggested using Stat-Ease 360 software based on the following desirability function. Maximizing drug solubility to reduce the total dosage of the formulation that needs to be administered to the patient. On the other hand, the phase transition temperature had to be close to 36 °C to guarantee transformation to a liquid state at body temperature. Moreover, it provides additional advantages by maximizing the safe margin to prevent undesirable conversion to the liquid state during storage. The composition of the optimized thermoresponsive solid dispersion formulation, the expected response values, and overall desirability are presented in Figure 4. The formulation composed of poloxamer 188, propylene glycol, and labrasol at concentrations of 13.89, 21.43, and 64.68% *w*/*w* is expected to have a solubility of 10.46 mg/g and a phase transition temperature of 35.81 °C.

### 3.10. Validation of Design

The optimized thermoresponsive solid dispersion was prepared to ensure that the response measurements fall within the expected values. This guarantees the present design’s ability to predict the interaction of formulation factors on the chosen responses within the selected range. Three independent formulations were prepared, and the measured responses are presented in Table 8. The mean solubility and phase transition temperature fall within the expected range, with a value of 10.68 mg/g and 36 °C, respectively.

### 3.11. Mechanism of Formulation Activation

**Figure 5:**
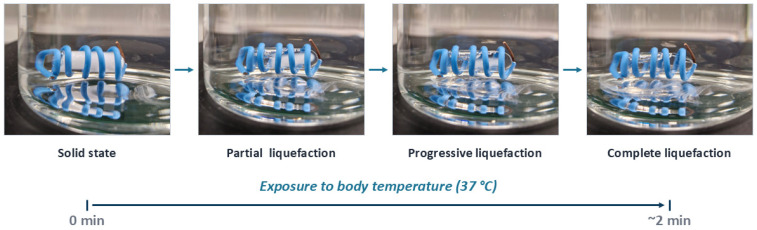
Phase transition of thermoresponsive solid dispersion formulation filled in a hard gelatin capsule during exposure to body temperature.

**Figure 6:**
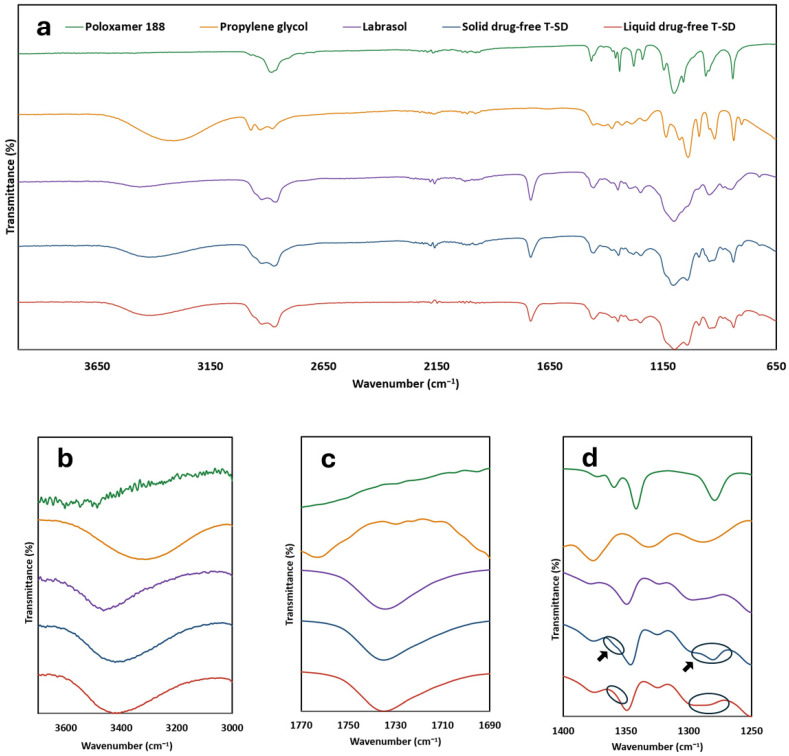
FTIR spectra of pure poloxamer 188, propylene glycol, and labrasol, and of the drug-free formulation in its solid and liquid states. (**a**) Full spectra (650–4000 cm^−1^); (**b**) O–H stretching region; (**c**) C=O ester carbonyl region; (**d**) CH_2_ wagging and twisting region.

The interaction of poloxamer 188, propylene glycol, and labrasol in a thermoresponsive solid dispersion formulation was studied using Fourier transform infrared (FTIR) spectroscopy. Figure 5 shows the thermoresponsive behavior of the formulation at physiological temperature (37 °C), which is above its phase transition temperature. Therefore, FTIR spectroscopy of the pure excipients and the drug-free formulation in both its solid and liquid states (Figure 6) was performed to provide a clear understanding of the molecular mechanism underlying the inverted thermal transition of the matrix. Poloxamer 188 spectrum showed a characteristic C–H stretching peak at 2882 cm^−1^, CH_2_ twisting at 1279 cm^−1^, CH_2_ wagging doublet at 1360 and 1342 cm^−1^, and C–O–C ether stretching at 1101 cm^−1^ [29,30]. Regarding propylene glycol, the reported intermolecular hydrogen bonding between the hydroxyl group is clearly indicated by a characteristic broad O–H stretching band at 3314 cm^−1^ [31]. Moreover, the C–O stretching peak was observed at 1039 cm^−1^, while C–H stretching was observed at 2971, 2931, and 2876 cm^−1^. Finally, the FTIR spectrum of labrasol showed characteristic C=O and C–O–C stretching peaks at 1734 and 1101 cm^−1^, respectively [32]. The thermal performance of a thermoresponsive solid dispersion is expected to arise from interactions among its excipients. Therefore, three distinct spectral regions were selected to explore interactions within the thermoresponsive solid dispersion. Figure 6 shows the highlighted spectral regions: the O–H stretching region (3000–3600 cm^−1^), the C=O ester carbonyl region (1700–1760 cm^−1^), and the CH_2_ wagging and twisting region (1250–1400 cm^−1^) corresponding to the crystalline 7/2 helical conformation of the poly(ethylene oxide) (PEO) terminal blocks of poloxamer 188.

In the O–H stretching region (Figure 6b), pure propylene glycol exhibited a broad band centered at 3314 cm^−1^, which is characteristic of intermolecular hydrogen bonding between the hydroxyl groups of propylene glycol [26]. In the drug-free formulation, the O–H stretching band appeared at 3422 cm^−1^ in the solid state and at 3429 cm^−1^ in the liquid state, substantially upshifted by more than 100 cm^−1^ relative to pure propylene glycol. These results provide clear evidence that propylene glycol hydroxyl groups have switched intermolecular hydrogen bonding from their hydroxyl oxygen to the weaker oxygen of poloxamer 188 [33]. Moreover, the preservation of the propylene-glycol CH_3_ bending band at 1376 cm^−1^ in pure agent and both formulation states (Figure 6d) further confirms that only the hydroxyl groups of propylene glycol participate in the matrix interactions. On the other hand, the ester carbonyl region (Figure 6c) was used to determine the role of labrasol in the hydrogen-bond network. The observed C=O stretching at 1734 cm^−1^ in the spectrum of pure labrasol is ascribed to the caprylocaproyl polyoxylglyceride esters in its structure. The retention of the C=O stretching peak in drug-free formulations, with no measurable shift between the solid and liquid states, suggests that the ester groups of labrasol do not participate in hydrogen bonding.

Pure poloxamer 188 displayed three markers of the crystalline 7/2 helical conformation of the PEO terminal blocks (Figure 6d): the CH_2_ wagging doublet at 1342 and 1360 cm^−1^ and the CH_2_ twisting band at 1279 cm^−1^ [30]. The preservation of all peaks in drug-free solid formulation indicates that the PEO 7/2 helical conformation preserves a significant degree of trans-conformational order in the solid state. On the contrary, the spectrum of the liquid state of formulation showed the disappearance of the 1360 cm^−1^ component of the CH_2_ wagging doublet and of the CH_2_ twisting band at 1279 cm^−1^. These observations indicate disruption of the trans conformation of the PEO 7/2 helix at the phase transition temperature and solubilization of poloxamer 188 and its transition from crystalline to amorphous solubilized state within the formulation [30]. The presence of poloxamer 188 at the same concentration in both the solid state and liquid state eliminates the possibility of masking effect by any other components. However, XRD was performed to assess the crystalline state of poloxamer in the solid state of the thermoresponsive solid dispersion formulation.

**Figure 7:**
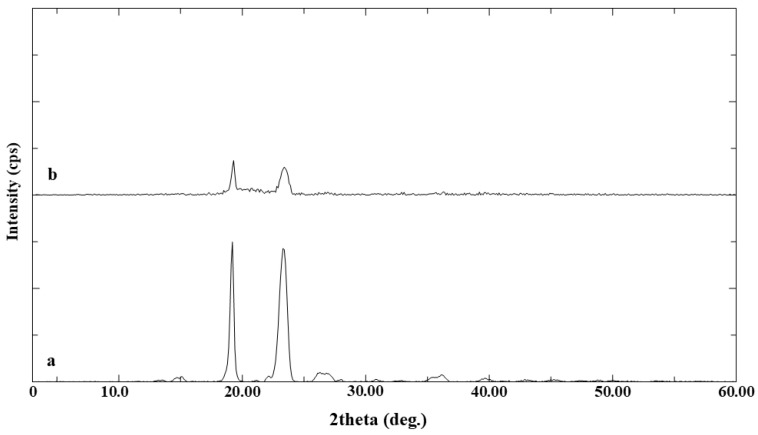
XRD pattern of (**a**) poloxamer and (**b**) solid drug-free thermoresponsive solid dispersion.

The XRD pattern of pure poloxamer 188 (Figure 7) showed two dominant sharp diffraction peaks at 2θ values of 19.2° and 23.3°, along with minor peaks at 26.3°, 27.0°, and 36.2°. These reflections arise exclusively from the PEO block segments of poloxamer 188, which is in agreement with previously reported X-ray diffractograms for poloxamer 188-based systems. On the other hand, the XRD pattern of the optimized thermoresponsive solid dispersion formulation retained diffraction peaks at the same angular positions (19.3° and 23.4° 2θ) with a dramatic reduction in intensities. The detected marked diminution of peak intensity without a shift in peak position reflects a substantial reduction in the volume fraction of crystalline PEO domains. However, it provides clear evidence of the preservation of the PEO block’s identical crystalline geometry, which agrees with the FTIR findings in the current study. This reduction in current results could be due to the two expected mechanisms. Firstly, the intercalation of propylene glycol molecules between poloxamer units via intermolecular hydrogen bonding disrupted the assembly of PEO helices. Secondly, the presence of labrasol could act as a diluent and plasticizer, altering the assembled crystalline structure of poloxamer and reducing its native nature.

Based on the FTIR and XRD findings, the mechanism of the solid-to-liquid thermal transition in the thermoresponsive solid dispersion formulation can be attributed to hydrogen bonding and residual poloxamer crystallinity. At room temperature, a solid state was attained by the formation of intermolecular hydrogen bonds between propylene glycol hydroxyl groups and ether oxygens of poloxamer 188 and the transformation of the PEO block to a crystalline state. Upon exposure to body temperature, hydrogen bonds are thermally disrupted, and the residual crystalline domains are solvated with propylene glycol.

### 3.12. In Vitro Dissolution

**Figure 8:**
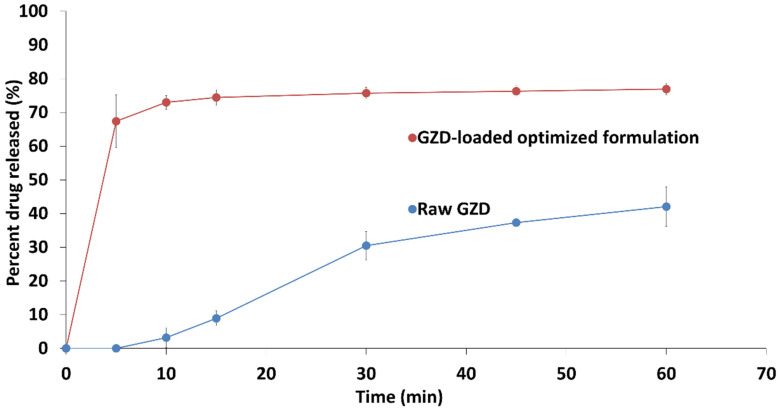
In vitro dissolution profile of raw gliclazide material and thermoresponsive solid dispersion.

An in vitro dissolution profile of gliclazide from capsules filled with raw drug and optimized thermoresponsive solid dispersion, and the obtained data were plotted in Figure 8. In addition, Table 9 shows the dissolution efficiency and initial dissolution rate. The result showed that the optimized formulation enhanced the drug’s initial dissolution rate by 8.4-fold compared to raw gliclazide. Moreover, there was a significant improvement in gliclazide dissolution efficiency, from 21.77 ± 4.74 to 74.85 ± 2.33, with the use of a thermoresponsive solid dispersion.

The observed augmentation in gliclazide dissolution with thermoresponsive solid dispersion could be attributed to several mechanisms. Firstly, rapid liquefaction and transition to the liquid state could result in a detectable increase in drug dissolution within the first 5 min, reaching 72.99 ± 2.07%. This resolves the aforementioned limitation of traditional solid dispersion formulations in the literature regarding drug entrapment within the formulation matrix. Moreover, the presence of the drug in a solubilized state within the formulation matrix could result in an outstanding initial dissolution rate compared to raw gliclazide. Furthermore, the expected formation of poloxamer 188 micelles provides a solubilization environment that enhances drug dissolution.

### 3.13. Characterization of Drug-Loaded Thermoresponsive Solid Dispersion

**Figure 9:**
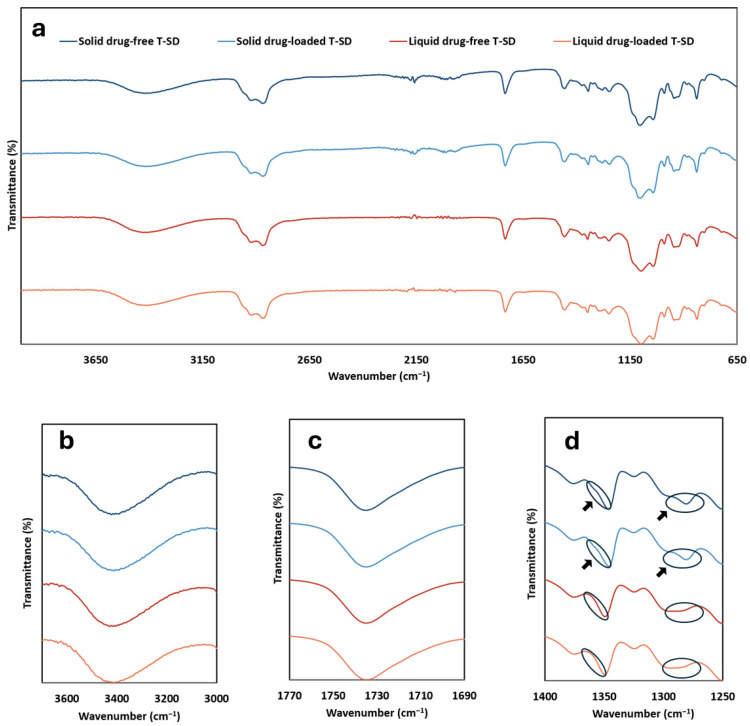
FTIR spectra of solid and liquid drug-free and drug-loaded thermoresponsive solid dispersions. (**a**) Full spectra (650–4000 cm^−1^); (**b**) O–H stretching region; (**c**) C=O ester carbonyl region; (**d**) CH_2_ wagging and twisting region.

The prepared drug-loaded thermoresponsive solid dispersion was assessed in two terms (impact of the drug on the thermoresponsive behavior of the solid dispersion and drug in the thermoresponsive solid dispersion). For the former, the pre-established three diagnostic regions were examined to test whether the matrix mechanism is altered by drug incorporation. The integration of gliclazide did not significantly affect the characteristic peaks in the solid and liquid states (Figure 9). In the hydroxyl and ester carbonyl region, both peaks were present, confirming that no interaction develops between gliclazide and the formed hydrogen bonding and the labrasol ester group, respectively. In addition, the disappearance of peaks at the CH2 wagging and twisting region indicates the preservation of thermoresponsive behavior of the solid dispersion. These observations establish that drug incorporation does not perturb the matrix mechanism characterized in Section 3.11.

**Figure 10:**
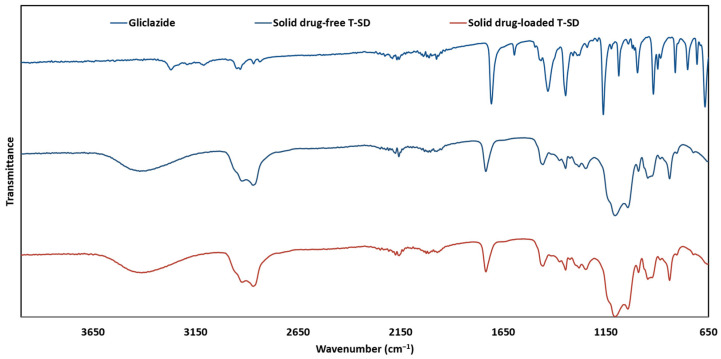
FTIR spectra of gliclazide and solid drug-free and drug-loaded thermoresponsive solid dispersions.

FTIR spectra of pure gliclazide, drug-free solid thermoresponsive solid dispersion, and the drug-loaded solid thermoresponsive solid dispersion were acquired (Figure 10). FTIR spectrum of gliclazide showed characteristic C=O stretch at 1708 cm^−1^ for the sulfonylurea, the S=O asymmetric and symmetric stretches at 1346 and 1162 cm^−1^, the aromatic/N–H bend at 1433 cm^−1^, and the N–H stretch at 3270 cm^−1^. The absence of gliclazide’s characteristic peaks in drug-loaded thermoresponsive solid dispersion could be ascribed to dilution and interference from formulation components. However, the crystalline nature of the drug was further examined by XRD to assess its physical state in formulations.

**Figure 11:**
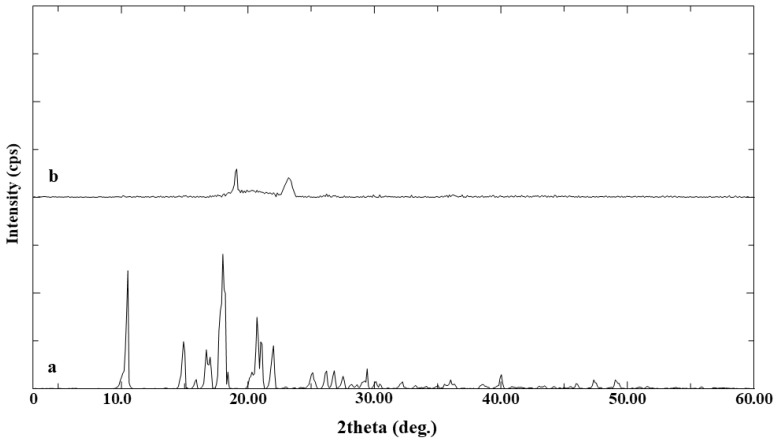
XRD pattern of (**a**) gliclazide and (**b**) solid drug-loaded thermoresponsive solid dispersion.

The X-ray diffraction pattern of crystalline gliclazide (Figure 11) showed multiple sharp reflections between 10° and 30° 2θ, in agreement with previously reported crystallographic data for the drug. The absence of characteristic peaks is a diagnostic signature of molecular dispersion in an amorphous solid-dispersion matrix [34]. Moreover, the alignment of observed crystalline peaks of poloxamer between drug-free and drug-loaded formulations provides clear evidence of the absence of drug effects on the thermoresponsive behavior of the thermoresponsive solid dispersion.

### 3.14. Green Assessment

The greenness of the thermoresponsive solid dispersion is ascribed to three distinct advantages over conventional solid dispersion technologies. First, avoiding organic solvents during its preparation eliminates residual solvent and the environmental concerns associated with solvent-based techniques such as spray drying and solvent evaporation. Second, the current approach minimizes energy consumption, whereas only mild heating is required to dissolve poloxamer in propylene glycol. Finally, eliminating the need for post-processing steps (pulverization, sieving, milling, or freeze-drying) overcomes the waste generation during the preparation of solid dispersion by other approaches. Therefore, these three advantages (solvent-free preparation, mild thermal processing, and absence of post-processing) reduce solvent consumption, energy demand, and waste generation.

## 4. Conclusions

The present study successfully developed a thermoresponsive solid dispersion formulation for gliclazide using the inverted thermoresponsive behavior of poloxamer 188 in propylene glycol. Surfactant screening identified labrasol as the optimal solubilizer, providing a 2.29-fold enhancement in gliclazide solubility compared to propylene glycol alone. The I-optimal mixture design effectively optimized the ternary composition, with the optimized formulation comprising poloxamer 188 (13.89% *w*/*w*), propylene glycol (21.43% *w*/*w*), and labrasol (64.68% *w*/*w*). Statistical analysis revealed that the synergistic interaction between propylene glycol and labrasol exerted a dominant influence on gliclazide solubility, while poloxamer 188 and propylene glycol significantly governed the phase transition temperature. The validated optimized formulation achieved a drug solubility of 10.68 mg/g and a phase transition temperature of 36 °C, confirming the predictive power of the design model. FTIR spectroscopy elucidated the mechanism of formulation activation, demonstrating that intermolecular hydrogen bonds between propylene glycol hydroxyl groups and poloxamer ether oxygens maintain the solid state at room temperature. Thermal disruption of these bonds at body temperature triggers complete liquefaction, enabling rapid drug release. The optimized formulation demonstrated an 8.4-fold increase in the initial dissolution rate and a significant improvement in dissolution efficiency, from 21.77 ± 4.74% to 74.85 ± 2.33% compared to raw gliclazide. The current approach eliminates the need for organic solvents, elevated processing temperatures, and post-processing steps, offering an industrially applicable alternative to conventional solid dispersion techniques.

## Figures and Tables

**Table 1:** Composition of the I-optimal mixture design formulations.

Run	Poloxamer 188 (*w*/*w*%)	Propylene Glycol (*w*/*w*%)	Labrasol (*w*/*w*%)
1	6.34	10.00	83.66
2	6.17	40.00	53.83
3	9.91	26.24	63.85
4	14.89	31.44	53.67
5	5.00	33.62	61.38
6	12.78	10.00	77.22
7	9.91	26.24	63.85
8	10.73	20.40	68.87
9	5.00	15.00	80.00
10	5.00	25.30	69.70
11	12.78	10.00	77.22
12	9.91	26.24	63.85
13	5.00	18.29	76.71
14	5.00	15.00	80.00
15	13.34	40.00	46.66
16	13.34	40.00	46.66
17	6.17	40.00	53.83

**Table 2:** Calibration curve characteristics of the developed UPLC method for the quantification of gliclazide.

Parameter	Value (Mean ± SD)
Concentration range (µg/mL)	1–75
Slope	0.1935 ± 0.0015
Intercept	0.043 ± 0.009
Coefficient of determination (R^2^)	0.9994 ± 0.0005
Standard error of regression	0.145 ± 0.050

**Table 3:** Solubility of gliclazide in propylene glycol–surfactant mixtures (1:1, *w*/*w*).

Medium	Solubility (mg/g)	Fold Improvement in Solubility
PG	4.59 ± 0.58	1.00
PG + HCO-30	7.00 ± 0.25	1.53
PG + Kolliphor EL	7.48 ± 0.20	1.63
PG + Tween 20	8.32 ± 0.09	1.81
PG + TO-10V	8.46 ± 0.33	1.84
PG + Tween 80	9.46 ± 0.36	2.06
PG + Labrasol	10.50 ± 0.13	2.29

**Table 4:** The measured responses of the I-optimal mixture design formulations.

Run	Solubility (mg/g)	Phase Transition Temp. (°C)
1	9.25	34
2	10.61	29
3	10.28	34
4	10.30	33
5	9.89	29
6	9.79	37
7	10.88	34
8	10.67	35
9	10.21	37
10	11.18	32
11	9.35	38
12	10.86	34
13	10.31	34
14	10.14	36
15	9.43	33
16	9.92	33
17	9.98	31

**Table 5:** Statistical validation parameters for the selected models.

Response	Model	Adjusted R^2^	Predicted R^2^	F-Value	*p*-Value	Lack of Fit (*p*-Value)
Solubility (mg/g)	Quadratic	0.5097	0.2241	4.33	0.0201	0.4009
Phase transition temperature (°C)	Linear	0.8868	0.8440	63.65	<0.0001	0.2846

**Table 6:** Analysis of variance (ANOVA) for the quadratic model fitted to gliclazide solubility.

Source	Sum of Squares	Mean Square	F-Value	*p*-Value
Model	3.24	0.6472	4.33	0.0201
Linear Mixture	0.3718	0.1859	1.24	0.3261
AB	0.0918	0.0918	0.6139	0.4499
AC	0.0765	0.0765	0.5111	0.4895
BC	2.73	2.73	18.24	0.0013

**Table 7:** Component effects (Cox and Piepel directions) for the phase transition temperature linear model.

Component	Component Effect (Cox)	Gradient Std Error	Approx t	Prob > |t|	Component Effect (Piepel)
Poloxamer 188 (A)	+4.15	5.65	7.34	<0.0001	+4.40
Propylene glycol (B)	−6.42	2.04	−10.52	<0.0001	−7.16
Labrasol (C)	+0.43	2.31	0.53	0.6020	+1.26

**Table 8:** Validation of the optimized formulation.

Analysis	Predicted Mean	Std Dev	n	SE Pred	95% PI Low	Data Mean	95% PI High
Solubility	10.46	0.39	3	0.28	9.84	10.68	11.07
Phase transition temperature	35.81	0.86	3	0.58	34.57	36.00	37.05

**Table 9:** Initial dissolution rate and dissolution efficiency of raw gliclazide material and thermoresponsive solid dispersion.

Test Agent	IDR (%/min)	DE (%)
Raw drug	0.59 ± 0.15	21.77 ± 4.74
Thermoresponsive solid dispersion	4.96 ± 0.16	74.85 ± 2.33

## Data Availability

The original contributions presented in this study are included in the article/supplementary material. Further inquiries can be directed to the corresponding author.

## Supplementary Materials

The following supporting information can be downloaded at: https://www.mdpi.com/article/10.3390/pharmaceutics18060702/s1, Figure S1: (a) Representative UPLC chromatogram of gliclazide. (b) Photodiode array (PDA) absorption spectrum of the gliclazide peak.

## Abbreviations

The following abbreviations are used in this manuscript:

ANOVA	Analysis of variance
DE	Dissolution efficiency
FTIR	Fourier transform infrared spectroscopy
HCO-30	Polyoxyl 30 hydrogenated castor oil
IDR	Initial dissolution rate
PEO	Poly(ethylene oxide)
PG	Propylene glycol
R^2^	Coefficient of determination
SD	Standard deviation
T-SD	Thermoresponsive solid dispersion
UPLC	Ultra-performance liquid chromatography
XRD	X-ray diffraction
