# Supplementary material for: Green Approach to Enhance Dissolution of Gliclazide: Thermoresponsive Solid Dispersion Based on Poloxamer 188/Propylene Glycol/Labrasol Ternary System
Source: Pharmaceutics. 2026 Jun 8;18(6):702. doi: 10.3390/pharmaceutics18060702 (PMC13306145; doi:10.3390/pharmaceutics18060702)
Supplement: Supplementary file 1 [file pharmaceutics-18-00702-s001.zip › pharmaceutics-4278648-supplementary.pdf]

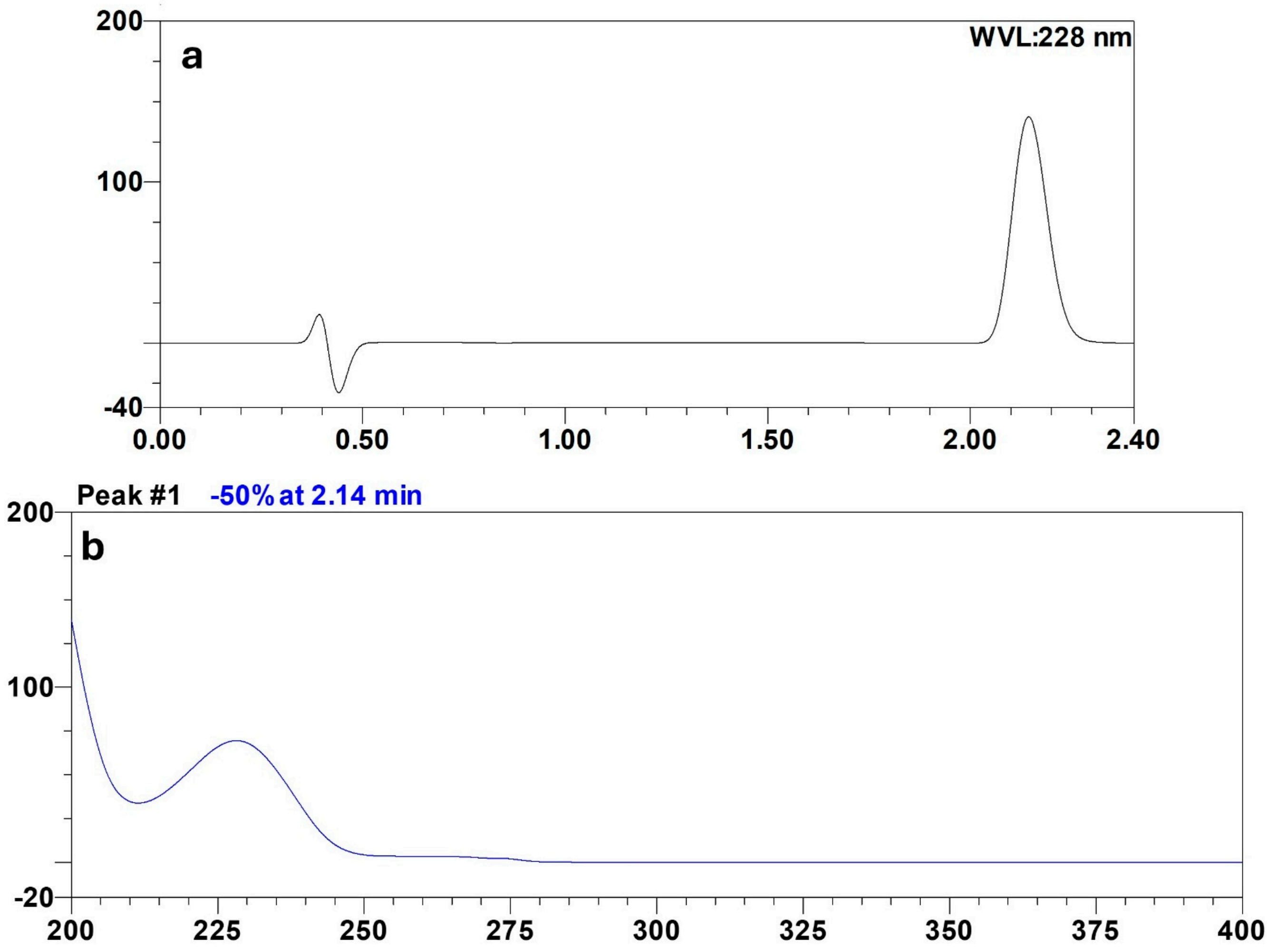

Figure S1. (a) Representative UPLC chromatogram of gliclazide. (b) Photodiode array (PDA) absorption spectrum of the gliclazide peak.
